# 1-Methyl-4-(4-methylstyryl)pyridinium 4-methylbenzenesulfonate

**DOI:** 10.1107/S1600536812044509

**Published:** 2012-11-03

**Authors:** M. Krishnakumar, S. Sudhahar, A. Silambarasan, G. Chakkaravarthi, R. Mohankumar

**Affiliations:** a Department of Physics, Presidency College, Chennai 600 005, India; b Department of Physics, CPCL Polytechnic College, Chennai 600 068, India; c Department of physics, Presidency College, Chennai 600 005, India

## Abstract

In the title salt, C_15_H_16_N^+^·C_7_H_7_O_3_S^−^, the dihedral angle between the pyridine and benzene rings of the cation is 5.98 (18)°. In the crystal, adjacent anions and cations are linked by weak non-classical C—H⋯O hydrogen bonds and π–π inter­actions, with a centroid–centroid distance of 3.749 (2) Å.

## Related literature

For mol­ecular compounds with non-linear optical properties, see: Bosshard *et al.* (1995 ▶); Nalwa & Miyata (1997 ▶). For related structures, see: Murugavel *et al.* (2009 ▶); Sivakumar *et al.* (2012 ▶); Okada *et al.* (1990 ▶).
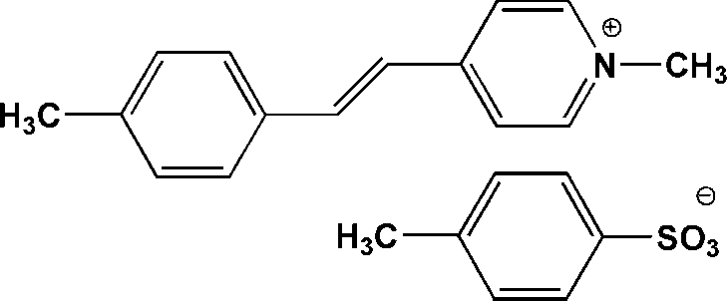


## Experimental

### 

#### Crystal data

C_15_H_16_N^+^·C_7_H_7_O_3_S^−^
*M* *_r_* = 381.48
Monoclinic, 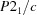

*a* = 9.1380 (6) Å
*b* = 6.4257 (5) Å
*c* = 33.884 (2) Å
β = 95.004 (4)°
*V* = 1982.0 (2) Å^3^
*Z* = 4
Mo *K*α radiation
μ = 0.19 mm^−1^
*T* = 295 K
0.28 × 0.22 × 0.20 mm

#### Data collection

Bruker Kappa APEXII diffractometer
Absorption correction: multi-scan (*SADABS*; Sheldrick, 1996 ▶) *T*_min_ = 0.950, *T*_max_ = 0.964
18512 measured reflections
4902 independent reflections
3850 reflections with *I* > 2σ(*I*)
*R*_int_ = 0.031

#### Refinement

*R*[*F* ^2^ > 2σ(*F* ^2^)] = 0.075
*wR*(*F* ^2^) = 0.184
*S* = 1.13
4902 reflections
247 parameters
1 restraint
H-atom parameters constrained
Δρ_max_ = 0.57 e Å^−3^
Δρ_min_ = −0.37 e Å^−3^

### 

Data collection: *APEX2* (Bruker, 2004 ▶); cell refinement: *SAINT* (Bruker, 2004 ▶); data reduction: *SAINT*; program(s) used to solve structure: *SHELXS97* (Sheldrick, 2008 ▶); program(s) used to refine structure: *SHELXL97* (Sheldrick, 2008 ▶); molecular graphics: *PLATON* (Spek, 2009 ▶); software used to prepare material for publication: *SHELXL97*.

## Supplementary Material

Crystal structure: contains datablock(s) global, I. DOI: 10.1107/S1600536812044509/rk2384sup1.cif

Structure factors: contains datablock(s) I. DOI: 10.1107/S1600536812044509/rk2384Isup2.hkl

Supplementary material file. DOI: 10.1107/S1600536812044509/rk2384Isup3.cml

Additional supplementary materials:  crystallographic information; 3D view; checkCIF report

## Figures and Tables

**Table 1:** Hydrogen-bond geometry (Å, °)

*D*—H⋯*A*	*D*—H	H⋯*A*	*D*⋯*A*	*D*—H⋯*A*
C3—H3⋯O2^i^	0.93	2.42	3.273 (4)	152
C14—H14*B*⋯O1^ii^	0.96	2.59	3.482 (4)	155

Symmetry codes: (i) 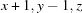; (ii) 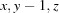.

**Fig. 1.**
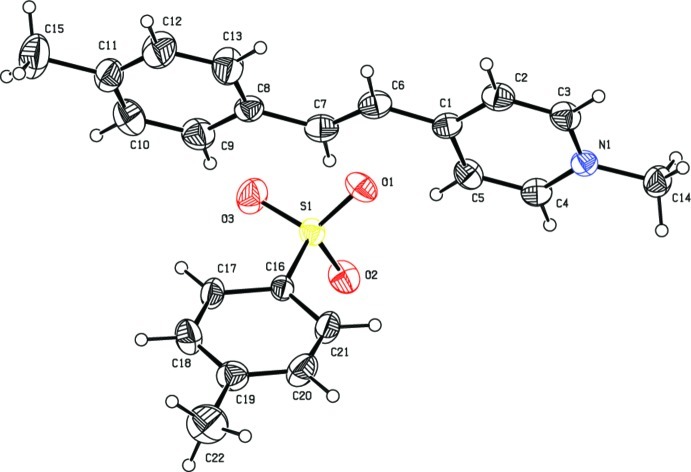
The molecular structure of I with atom labels. Displacement ellipsoids are drawn at 30% probability level. H atoms are presented as a small spheres of arbitrary radius.

**Fig. 2.**
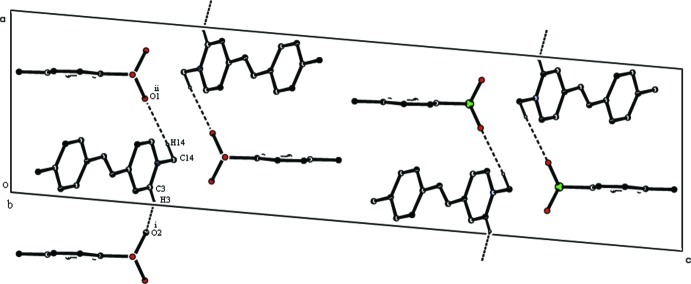
The packing of I, viewed down b axis. Intermolecular hydrogen bonds are shown as dashed lines. H atoms not involved in hydrogen bonding have been omitted for clarity.

## supplementary crystallographic information

### Comment

In continuation of our studies of molecular compounds with non linear optical properties which are known to exhibit applications in optoelectronic and photonic devices (Bosshard *et al.*, 1995; Nalwa & Miyata, 1997), we determined the crystal structure of the title compound I.

The asymmetric unit of I, (Fig. 1), contains C_15_H_16_N^+^ cation and C_7_H_7_O_3_S^-^ anion. The geometric parameters of the title compound are comparable with the similar reported structures: Murugavel *et al.*, 2009; Sivakumar *et al.*, 2012; Okada *et al.*, 1990. The cation is planar - torsion angle about the double bond between the two rings in the cation, C1–C6═C7–C8 is 178.2 (3)°. The benzene ring in the anion is almost planar, with the maximum deviation of 0.003 (3)Å.

In the crystal structure, the adjacent anions and cations are linked by weak non-classical C–H···O H bonds (Table 1 & Fig.2) and π–π interactions - Cg1···Cg2^iii^ = 3.749 (2)Å, where Cg1 and Cg2 are the centroids of the rings (C1-C5/N1) and (C8-C13), respectively. Symmetry code: (iii) *x*, -*y*-1/2, *z*+1/2.

### Experimental

The title compound was synthesized by the condensation of 4-methyl-*N*-methyl pyridinium tosylate, which was prepared from 4-picoline (4.65 g, 5 mmol) and methyl *p*-toluenesulfonate (9.31 g, 5 mmol), and 4-methylbenzaldehyde (6 g, 5 mmol) in the presence of piperidine. The single crystals were grown by slow evaporation method in room temperature.

### Refinement

The H atoms were positioned geometrically and refined using riding model with C–H = 0.93Å and *U*_iso_(H) = 1.2*U*_eq_(C) for aromatic H, C–H = 0.96Å and *U*_iso_(H) = 1.5*U*_eq_(C) for methyl H. The components of the anisotropic displacement parameters for C7 and C8 were restrained to be equal within an effective deviation of 0.001 using *DELU* instruction in *SHELXL* (Sheldrick, 2008).

### Crystal data

**Table:**

C_15_H_16_N^+^·C_7_H_7_O_3_S^−^	*F*(000) = 808
*M**_r_* = 381.48	*D*_x_ = 1.278 Mg m^−^^3^
Monoclinic, *P*2_1_/*c*	Mo *K*α radiation, λ = 0.71073 Å
Hall symbol: -P 2ybc	Cell parameters from 4112 reflections
*a* = 9.1380 (6) Å	θ = 2.2–28.4°
*b* = 6.4257 (5) Å	µ = 0.19 mm^−^^1^
*c* = 33.884 (2) Å	*T* = 295 K
β = 95.004 (4)°	Block, colourless
*V* = 1982.0 (2) Å^3^	0.28 × 0.22 × 0.20 mm
*Z* = 4	

### Data collection

**Table:**

Bruker Kappa APEXII diffractometer	4902 independent reflections
Radiation source: fine-focus sealed tube	3850 reflections with *I* > 2σ(*I*)
Graphite monochromator	*R*_int_ = 0.031
ω– and φ–scans	θ_max_ = 28.4°, θ_min_ = 2.2°
Absorption correction: multi-scan (*SADABS*; Sheldrick, 1996)	*h* = −12→12
*T*_min_ = 0.950, *T*_max_ = 0.964	*k* = −8→8
18512 measured reflections	*l* = −44→45

### Refinement

**Table:**

Refinement on *F*^2^	Primary atom site location: structure-invariant direct methods
Least-squares matrix: full	Secondary atom site location: difference Fourier map
*R*[*F*^2^ > 2σ(*F*^2^)] = 0.075	Hydrogen site location: inferred from neighbouring sites
*wR*(*F*^2^) = 0.184	H-atom parameters constrained
*S* = 1.13	*w* = 1/[σ^2^(*F*_o_^2^) + (0.0478*P*)^2^ + 2.5149*P*] where *P* = (*F*_o_^2^ + 2*F*_c_^2^)/3
4902 reflections	(Δ/σ)_max_ < 0.001
247 parameters	Δρ_max_ = 0.57 e Å^−^^3^
1 restraint	Δρ_min_ = −0.37 e Å^−^^3^

### Special details

**Table:**

Geometry. All s.u.'s (except the s.u. in the dihedral angle between two l.s. planes) are estimated using the full covariance matrix. The cell s.u.'s are taken into account individually in the estimation of s.u.'s in distances, angles and torsion angles; correlations between s.u.'s in cell parameters are only used when they are defined by crystal symmetry. An approximate (isotropic) treatment of cell s.u.'s is used for estimating s.u.'s involving l.s. planes.
Refinement. Refinement of *F*^2^ against ALL reflections. The weighted *R*-factor w*R* and goodness of fit *S* are based on *F*^2^, conventional *R*-factors *R* are based on *F*, with *F* set to zero for negative *F*^2^. The threshold expression of *F*^2^ > 2σ(*F*^2^) is used only for calculating *R*-factors(gt) etc. and is not relevant to the choice of reflections for refinement. *R*-factors based on *F*^2^ are statistically about twice as large as those based on *F*, and *R*-factors based on ALL data will be even larger.

### Fractional atomic coordinates and isotropic or equivalent isotropic displacement parameters (Å^2^)

**Table:**

	*x*	*y*	*z*	*U*_iso_*/*U*_eq_	
C1	0.8177 (3)	0.1727 (5)	0.32460 (9)	0.0480 (7)	
C2	0.9323 (3)	0.0485 (6)	0.31430 (10)	0.0586 (9)	
H2	1.0286	0.0873	0.3219	0.070*	
C3	0.9059 (3)	−0.1293 (6)	0.29319 (10)	0.0557 (8)	
H3	0.9848	−0.2100	0.2867	0.067*	
C4	0.6546 (3)	−0.0742 (5)	0.29074 (9)	0.0519 (7)	
H4	0.5595	−0.1168	0.2826	0.062*	
C5	0.6764 (3)	0.1060 (5)	0.31182 (10)	0.0535 (8)	
H5	0.5959	0.1851	0.3177	0.064*	
C6	0.8525 (4)	0.3602 (6)	0.34869 (10)	0.0581 (8)	
H6	0.9514	0.3895	0.3552	0.070*	
C7	0.7580 (4)	0.4879 (6)	0.36164 (10)	0.0562 (8)	
H7	0.6593	0.4607	0.3545	0.067*	
C8	0.7923 (4)	0.6722 (5)	0.38662 (9)	0.0511 (7)	
C9	0.6807 (4)	0.8026 (7)	0.39551 (11)	0.0687 (10)	
H9	0.5849	0.7725	0.3856	0.082*	
C10	0.7073 (5)	0.9779 (7)	0.41889 (13)	0.0780 (12)	
H10	0.6288	1.0626	0.4242	0.094*	
C11	0.8446 (5)	1.0290 (6)	0.43424 (10)	0.0666 (10)	
C12	0.9570 (5)	0.8987 (8)	0.42589 (13)	0.0834 (13)	
H12	1.0523	0.9282	0.4363	0.100*	
C13	0.9312 (4)	0.7257 (7)	0.40249 (13)	0.0757 (11)	
H13	1.0101	0.6419	0.3972	0.091*	
C14	0.7436 (4)	−0.3798 (5)	0.25756 (10)	0.0623 (9)	
H14A	0.7213	−0.3417	0.2303	0.093*	
H14B	0.6626	−0.4558	0.2666	0.093*	
H14C	0.8301	−0.4652	0.2600	0.093*	
C15	0.8745 (7)	1.2200 (7)	0.45951 (14)	0.1050 (18)	
H15A	0.7848	1.2962	0.4611	0.157*	
H15B	0.9449	1.3065	0.4479	0.157*	
H15C	0.9127	1.1789	0.4856	0.157*	
C16	0.3056 (3)	0.4990 (4)	0.36655 (8)	0.0397 (6)	
C17	0.3040 (4)	0.6399 (5)	0.39722 (10)	0.0530 (8)	
H17	0.2963	0.7815	0.3917	0.064*	
C18	0.3140 (4)	0.5713 (6)	0.43625 (10)	0.0669 (10)	
H18	0.3134	0.6685	0.4566	0.080*	
C19	0.3247 (4)	0.3641 (6)	0.44559 (11)	0.0619 (9)	
C20	0.3257 (5)	0.2259 (6)	0.41467 (12)	0.0685 (10)	
H20	0.3332	0.0845	0.4203	0.082*	
C21	0.3159 (4)	0.2887 (5)	0.37551 (11)	0.0587 (8)	
H21	0.3163	0.1907	0.3553	0.070*	
C22	0.3344 (6)	0.2865 (9)	0.48800 (12)	0.0999 (16)	
H22A	0.2391	0.2933	0.4978	0.150*	
H22B	0.4019	0.3719	0.5041	0.150*	
H22C	0.3684	0.1450	0.4889	0.150*	
N1	0.7693 (3)	−0.1908 (4)	0.28160 (7)	0.0447 (6)	
O1	0.4209 (2)	0.5016 (4)	0.30040 (7)	0.0603 (6)	
O2	0.1565 (2)	0.5055 (4)	0.29878 (7)	0.0611 (6)	
O3	0.2956 (3)	0.8137 (4)	0.31892 (8)	0.0753 (8)	
S1	0.29376 (8)	0.58894 (12)	0.31685 (2)	0.0444 (2)	

### Atomic displacement parameters (Å^2^)

**Table:**

	*U*^11^	*U*^22^	*U*^33^	*U*^12^	*U*^13^	*U*^23^
C1	0.0485 (16)	0.0467 (17)	0.0488 (16)	−0.0024 (13)	0.0038 (13)	0.0100 (13)
C2	0.0406 (15)	0.070 (2)	0.065 (2)	−0.0036 (15)	0.0044 (14)	0.0021 (18)
C3	0.0442 (16)	0.058 (2)	0.066 (2)	0.0095 (15)	0.0092 (14)	0.0037 (16)
C4	0.0411 (15)	0.0583 (19)	0.0555 (17)	0.0006 (14)	−0.0001 (13)	0.0049 (16)
C5	0.0463 (16)	0.0573 (19)	0.0573 (17)	0.0138 (15)	0.0063 (13)	0.0017 (16)
C6	0.0443 (16)	0.063 (2)	0.067 (2)	−0.0036 (15)	0.0009 (15)	0.0071 (17)
C7	0.0491 (17)	0.0618 (19)	0.0572 (18)	−0.0059 (15)	0.0013 (14)	0.0045 (14)
C8	0.0536 (17)	0.0527 (17)	0.0471 (15)	−0.0037 (14)	0.0054 (13)	0.0111 (12)
C9	0.0517 (19)	0.081 (3)	0.073 (2)	−0.0037 (19)	0.0066 (17)	−0.006 (2)
C10	0.077 (3)	0.083 (3)	0.077 (3)	0.018 (2)	0.017 (2)	−0.007 (2)
C11	0.098 (3)	0.056 (2)	0.0452 (17)	−0.007 (2)	0.0031 (18)	−0.0002 (16)
C12	0.064 (2)	0.094 (3)	0.090 (3)	−0.015 (2)	−0.007 (2)	−0.023 (3)
C13	0.055 (2)	0.077 (3)	0.094 (3)	0.0079 (19)	−0.0004 (19)	−0.020 (2)
C14	0.086 (3)	0.0438 (18)	0.0580 (19)	0.0003 (17)	0.0103 (17)	0.0020 (15)
C15	0.172 (5)	0.071 (3)	0.070 (3)	−0.007 (3)	0.004 (3)	−0.016 (2)
C16	0.0302 (12)	0.0407 (14)	0.0484 (14)	0.0002 (11)	0.0045 (10)	−0.0091 (12)
C17	0.0646 (19)	0.0361 (15)	0.0587 (18)	0.0034 (14)	0.0073 (15)	−0.0088 (14)
C18	0.082 (2)	0.069 (2)	0.0492 (18)	0.005 (2)	0.0062 (17)	−0.0137 (18)
C19	0.063 (2)	0.067 (2)	0.0559 (19)	−0.0052 (17)	0.0040 (16)	0.0039 (17)
C20	0.088 (3)	0.0448 (18)	0.071 (2)	−0.0081 (18)	0.000 (2)	0.0052 (18)
C21	0.075 (2)	0.0398 (17)	0.0613 (19)	−0.0021 (16)	0.0046 (16)	−0.0086 (15)
C22	0.126 (4)	0.109 (4)	0.063 (2)	−0.008 (3)	0.000 (3)	0.017 (3)
N1	0.0504 (13)	0.0415 (13)	0.0426 (12)	0.0024 (11)	0.0059 (10)	0.0067 (11)
O1	0.0446 (12)	0.0814 (17)	0.0565 (13)	0.0081 (11)	0.0131 (10)	−0.0025 (12)
O2	0.0406 (11)	0.0816 (17)	0.0599 (13)	0.0075 (11)	−0.0034 (9)	−0.0081 (12)
O3	0.109 (2)	0.0446 (13)	0.0723 (16)	0.0049 (14)	0.0096 (15)	0.0082 (12)
S1	0.0406 (4)	0.0440 (4)	0.0488 (4)	0.0053 (3)	0.0045 (3)	−0.0026 (3)

### Geometric parameters (Å, º)

**Table:**

C1—C2	1.385 (5)	C14—N1	1.470 (4)
C1—C5	1.393 (4)	C14—H14A	0.9600
C1—C6	1.474 (5)	C14—H14B	0.9600
C2—C3	1.358 (5)	C14—H14C	0.9600
C2—H2	0.9300	C15—H15A	0.9600
C3—N1	1.336 (4)	C15—H15B	0.9600
C3—H3	0.9300	C15—H15C	0.9600
C4—N1	1.346 (4)	C16—C17	1.379 (4)
C4—C5	1.366 (5)	C16—C21	1.386 (4)
C4—H4	0.9300	C16—S1	1.775 (3)
C5—H5	0.9300	C17—C18	1.389 (5)
C6—C7	1.296 (5)	C17—H17	0.9300
C6—H6	0.9300	C18—C19	1.370 (5)
C7—C8	1.474 (5)	C18—H18	0.9300
C7—H7	0.9300	C19—C20	1.374 (5)
C8—C9	1.373 (5)	C19—C22	1.517 (5)
C8—C13	1.378 (5)	C20—C21	1.383 (5)
C9—C10	1.386 (6)	C20—H20	0.9300
C9—H9	0.9300	C21—H21	0.9300
C10—C11	1.357 (6)	C22—H22A	0.9600
C10—H10	0.9300	C22—H22B	0.9600
C11—C12	1.373 (6)	C22—H22C	0.9600
C11—C15	1.507 (6)	O1—S1	1.446 (2)
C12—C13	1.374 (6)	O2—S1	1.449 (2)
C12—H12	0.9300	O3—S1	1.446 (3)
C13—H13	0.9300		
			
C2—C1—C5	116.4 (3)	N1—C14—H14C	109.5
C2—C1—C6	118.6 (3)	H14A—C14—H14C	109.5
C5—C1—C6	124.9 (3)	H14B—C14—H14C	109.5
C3—C2—C1	120.9 (3)	C11—C15—H15A	109.5
C3—C2—H2	119.5	C11—C15—H15B	109.5
C1—C2—H2	119.5	H15A—C15—H15B	109.5
N1—C3—C2	121.5 (3)	C11—C15—H15C	109.5
N1—C3—H3	119.3	H15A—C15—H15C	109.5
C2—C3—H3	119.3	H15B—C15—H15C	109.5
N1—C4—C5	120.7 (3)	C17—C16—C21	118.7 (3)
N1—C4—H4	119.6	C17—C16—S1	119.8 (2)
C5—C4—H4	119.6	C21—C16—S1	121.5 (2)
C4—C5—C1	120.8 (3)	C16—C17—C18	120.3 (3)
C4—C5—H5	119.6	C16—C17—H17	119.9
C1—C5—H5	119.6	C18—C17—H17	119.9
C7—C6—C1	125.9 (3)	C19—C18—C17	121.7 (3)
C7—C6—H6	117.0	C19—C18—H18	119.1
C1—C6—H6	117.0	C17—C18—H18	119.1
C6—C7—C8	126.1 (3)	C18—C19—C20	117.2 (3)
C6—C7—H7	117.0	C18—C19—C22	122.4 (4)
C8—C7—H7	117.0	C20—C19—C22	120.4 (4)
C9—C8—C13	116.0 (3)	C19—C20—C21	122.6 (3)
C9—C8—C7	119.5 (3)	C19—C20—H20	118.7
C13—C8—C7	124.5 (3)	C21—C20—H20	118.7
C8—C9—C10	121.7 (4)	C20—C21—C16	119.5 (3)
C8—C9—H9	119.2	C20—C21—H21	120.3
C10—C9—H9	119.2	C16—C21—H21	120.3
C11—C10—C9	121.7 (4)	C19—C22—H22A	109.5
C11—C10—H10	119.1	C19—C22—H22B	109.5
C9—C10—H10	119.1	H22A—C22—H22B	109.5
C10—C11—C12	117.2 (4)	C19—C22—H22C	109.5
C10—C11—C15	122.0 (4)	H22A—C22—H22C	109.5
C12—C11—C15	120.8 (4)	H22B—C22—H22C	109.5
C11—C12—C13	121.2 (4)	C3—N1—C4	119.6 (3)
C11—C12—H12	119.4	C3—N1—C14	120.5 (3)
C13—C12—H12	119.4	C4—N1—C14	119.8 (3)
C12—C13—C8	122.2 (4)	O1—S1—O3	113.58 (17)
C12—C13—H13	118.9	O1—S1—O2	112.85 (14)
C8—C13—H13	118.9	O3—S1—O2	113.34 (17)
N1—C14—H14A	109.5	O1—S1—C16	104.78 (13)
N1—C14—H14B	109.5	O3—S1—C16	106.24 (15)
H14A—C14—H14B	109.5	O2—S1—C16	105.02 (14)
			
C5—C1—C2—C3	−0.6 (5)	C21—C16—C17—C18	0.6 (5)
C6—C1—C2—C3	178.0 (3)	S1—C16—C17—C18	−179.7 (3)
C1—C2—C3—N1	0.0 (5)	C16—C17—C18—C19	−0.4 (6)
N1—C4—C5—C1	−0.4 (5)	C17—C18—C19—C20	0.2 (6)
C2—C1—C5—C4	0.8 (5)	C17—C18—C19—C22	−179.5 (4)
C6—C1—C5—C4	−177.7 (3)	C18—C19—C20—C21	−0.2 (6)
C2—C1—C6—C7	−177.8 (3)	C22—C19—C20—C21	179.5 (4)
C5—C1—C6—C7	0.6 (6)	C19—C20—C21—C16	0.4 (6)
C1—C6—C7—C8	178.2 (3)	C17—C16—C21—C20	−0.6 (5)
C6—C7—C8—C9	174.9 (4)	S1—C16—C21—C20	179.6 (3)
C6—C7—C8—C13	−5.5 (6)	C2—C3—N1—C4	0.4 (5)
C13—C8—C9—C10	0.4 (6)	C2—C3—N1—C14	177.8 (3)
C7—C8—C9—C10	180.0 (4)	C5—C4—N1—C3	−0.2 (5)
C8—C9—C10—C11	−0.2 (7)	C5—C4—N1—C14	−177.6 (3)
C9—C10—C11—C12	−0.4 (6)	C17—C16—S1—O1	124.9 (2)
C9—C10—C11—C15	179.7 (4)	C21—C16—S1—O1	−55.3 (3)
C10—C11—C12—C13	0.9 (7)	C17—C16—S1—O3	4.4 (3)
C15—C11—C12—C13	−179.2 (4)	C21—C16—S1—O3	−175.8 (3)
C11—C12—C13—C8	−0.8 (7)	C17—C16—S1—O2	−116.0 (3)
C9—C8—C13—C12	0.1 (6)	C21—C16—S1—O2	63.8 (3)
C7—C8—C13—C12	−179.5 (4)		

### Hydrogen-bond geometry (Å, º)

**Table:**

*D*—H···*A*	*D*—H	H···*A*	*D*···*A*	*D*—H···*A*
C3—H3···O2^i^	0.93	2.42	3.273 (4)	152
C14—H14*B*···O1^ii^	0.96	2.59	3.482 (4)	155

Symmetry codes: (i) *x*+1, *y*−1, *z*; (ii) *x*, *y*−1, *z*.
